# Intravenous umbilical cord-derived mesenchymal stromal cell therapy may improve overall survival in Japanese patients with idiopathic pneumonia syndrome after hematopoietic stem cell transplantation: a multicenter, single-arm, phase II trial

**DOI:** 10.1007/s12185-025-04024-x

**Published:** 2025-07-15

**Authors:** Noriko Doki, Nobuharu Fujii, Shinichi Kako, Emiko Sakaida, Yoshinobu Kanda

**Affiliations:** 1 Hematology Division, Tokyo Metropolitan Cancer and Infectious Diseases Center, Komagome Hospital, Bunkyo-Ku, Tokyo Japan; 2 Department of Hematology and Oncology, Okayama University Hospital, Kita-Ku, Okayama, Okayama Japan; 3 Division of Hematology, Jichi Medical University Saitama Medical Center, Omiya-Ku, Saitama, Saitama Japan; 4 Department of Hematology, Chiba University Hospital, Chiba, Chiba Japan; 5 Division of Hematology, Department of Medicine, Jichi Medical University, Shimotsuke, Tochigi Japan

**Keywords:** Graft-versus-host disease, Hematopoietic stem cell transplantation, Idiopathic pneumonia syndrome, Overall survival, Umbilical cord-derived mesenchymal stromal cells

## Abstract

Idiopathic pneumonia syndrome (IPS) is a serious complication of allogeneic hematopoietic stem cell transplantation (HSCT) and has a poor prognosis. Although IPS is often treated with steroids, the disease can become resistant to or dependent on steroid treatment, and there is no effective cure for patients with refractory or steroid-dependent IPS. This multicenter, open-label, single-arm, phase II clinical trial investigated the efficacy and safety of HLC-001 (allogeneic umbilical cord-derived mesenchymal stromal cells) in patients with progressive steroid-dependent or refractory IPS after HSCT. Seven male patients (all male; mean age: 43.3 years) received HLC-001 and three completed the trial. The survival rate at day 56 (primary endpoint) was 71.4% (5/7 patients; 95% confidence interval: 29.0%–96.3%) and was sustained at day 100, suggesting that HLC-001 was more effective than previously reported treatment. Three of the five patients with ≥ 100 days of follow-up died. Five patients experienced at least one adverse drug reaction, none of which were serious. These findings indicate that HLC-001 was potentially effective and generally well tolerated in Japanese patients with steroid-dependent or refractory IPS after HSCT. Given there is no effective cure for steroid-dependent or refractory IPS, HLC-001 may be a promising treatment option and further clinical evaluation is warranted.

Trial registration: Japan Registry of Clinical Trials identifier: jRCT2063220014.

**Supplementary Information:**

The online version contains supplementary material available at 10.1007/s12185-025-04024-x.

## Introduction

Allogeneic hematopoietic stem cell transplantation (HSCT) can result in serious complications with poor prognoses, including idiopathic pneumonia syndrome (IPS) [1]. IPS after allogeneic HSCT has a reported incidence of 3.7%–12.0%, and is commonly treated with steroid therapy [2–5]. However, the condition can be difficult to control and may become steroid-dependent or resistant to treatment. Multiple studies have indicated that the inflammatory cytokine tumor necrosis factor (TNF)-α plays a significant role in the onset and progression of IPS [6, 7]. A phase III, randomized, double-blind, placebo-controlled trial evaluating the efficacy of etanercept, a soluble TNF receptor, in treating IPS was conducted; however, it did not achieve its primary endpoints, failing to demonstrate significant efficacy. [8]. Neither steroid-dependent nor refractory IPS have an effective cure and are associated with rapid deterioration and a high mortality rate [1, 2, 7, 9]. Therefore, there is a need for new, effective treatment options for progressive steroid-dependent and refractory IPS.

IPS has clinical characteristics that are very similar to graft-versus-host disease (GVHD) [10]. Mesenchymal stromal cells (MSCs) have been reported to have therapeutic effects against these conditions. Among MSCs, umbilical cord-derived MSCs have advantages over MSCs derived from other tissues: they can be harvested from tissues that are usually discarded without causing any harm or physical burden to the donor, and they have a high proliferative capacity [11, 12]. Their potential therapeutic uses have been investigated in a variety of conditions, including GVHD, hematologic disorders, neurological disorders, diabetes mellitus, and other diseases [11, 12].

HLC-001 is an allogeneic umbilical cord-derived MSC product manufactured by the Institute of Medical Science (University of Tokyo) [13]. When activated by inflammatory cytokines (such as interferon-γ and TNF-α) at the site of inflammation or tissue damage, these cells exert an anti-inflammatory effect and contribute to tissue repair [13].

HLC-001 treatment has been shown to be effective and well tolerated in patients with acute GVHD [13]. As mentioned above, because the clinical conditions of IPS are very similar to those of GVHD and acute respiratory distress syndrome, HLC-001 may be an effective treatment for IPS after HSCT. However, the effects of umbilical cord-derived MSCs on IPS are still unknown.

We conducted this trial to investigate the efficacy and safety of HLC-001 treatment in Japanese patients with progressive steroid-dependent or refractory IPS after HSCT and report the results herein. Given the absence of effective treatments for these patients and their poor prognosis, there was no placebo control in this phase II trial.

## Methods

### Study design and treatment

This multicenter, open-label, single-arm, phase II clinical trial was conducted in Japan. Patients (≥ 16 years) with progressive steroid-dependent or refractory IPS after HSCT were enrolled between October 2022 and June 2023. IPS was defined based on a statement from the American Thoracic Society [7]. Briefly, IPS was defined as the presence of widespread alveolar injury confirmed by chest imaging; absence of lower respiratory tract infection, as determined by bronchoalveolar lavage (BAL); and no identifiable cardiac, renal, or iatrogenic etiology. Corticosteroid-treated IPS (≥ 1 mg/kg/day prednisolone or equivalent) that deteriorated after 3 days of treatment, or did not improve after 5 days of treatment, was defined as steroid-dependent/refractory IPS. All patients were required to undergo bronchoscopy with BAL at trial entry. The detailed study design is described in the Supplementary Methods.

Patients received HLC-001 intravenously at a dose of 2 × 10^6^ cells/kg, once daily, for two doses per cycle at intervals between 2 and 7 days, for a maximum of four cycles and eight doses. The dose of HLC-001 was determined based on the results of a phase I trial of HLC-001 in patients with acute GVHD [13]. This dose was tolerated in the previous study and is equivalent to the clinical dose of Temcell^®^ HS (JCR Pharmaceuticals Co., Ltd., Ashiya, Japan) injection, which is a bone marrow-derived mesenchymal stem cell treatment.

The trial was approved at participating sites (Table S1) by the relevant institutional review boards, registered with the Japan Registry of Clinical Trials (jRCT2063220014), and conducted in accordance with the principles of the Declaration of Helsinki, Good Clinical Practice, and applicable local regulations. Patients or their legal guardian provided written informed consent prior to enrollment.

### Study endpoints

The primary endpoint was overall survival at 56 days after treatment initiation. Secondary endpoints were overall survival at day 100; percentage of patients not undergoing mechanical ventilation at days 28, 56, and 100; number of days without mechanical ventilation from treatment initiation to days 28, 56, and 100; percentage of patients who did not require an increase in oxygen dose, percentage of patients not requiring oxygen treatment and percentage of patients with oxygen dose reduced by ≥ 50% on days 28, 56, and 100; number of days in which increased oxygen administration was not required, time period during which oxygen administration was not required and period during which the reduction in oxygen dose by ≥ 50% was achieved on days 28, 56, and 100; percentage of patients with corticosteroid dose decreased to < 1 mg/kg/day and the amount reduced; partial pressure of oxygen in arterial blood (PaO_2_) and fraction of inspired oxygen (FiO_2_) ratio; evaluation of chest X-ray/computed tomography (CT) scans; Eastern Cooperative Oncology Group performance status (ECOG-PS) scores [14]; surfactant protein-D (SP-D) and Krebs von den Lungen-6 (KL-6) levels at each time point up to day 100; and adverse events (AEs) and their severity according to the Common Terminology Criteria for Adverse Events. Exploratory endpoints included changes in cytokines, chemokines, and immune cells before each dose and on days 28 and 56. Further details are described in the Supplementary Methods.

### Statistical methods

This analysis was conducted based on the Thall & Simon Bayesian design (TS design) [15] (described in the Supplementary Methods). A sensitivity analysis was performed for the primary endpoint, including calculation of survival rates and their 95% Clopper–Pearson confidence intervals (CIs) and the 95% highest posterior density intervals for the posterior distribution of the survival probability of the treatment under an uninformed prior distribution. According to the TS design criteria, the minimum number of survivors required to indicate that HLC-001 was more effective than existing therapies is summarized in Table 1. The planned sample size ranged from a minimum of six to a maximum of twenty patients. Summary statistics were calculated for patient backgrounds and secondary endpoints. Statistical analyses were conducted using R Statistical Software (v4.3.0) [16].

**Table 1:** Minimum number of survivors required to demonstrate efficacy (Thall & Simon Bayesian design)

Enrolled number	6	7	8	9	10	11	12	13	14	15	16	17	18	19	20
Minimum number of survivors	5	5	5	6	6	6	7	7	8	8	8	9	9	9	10

## Results

### Patients and HLC-001 treatment

**Fig. 1:**
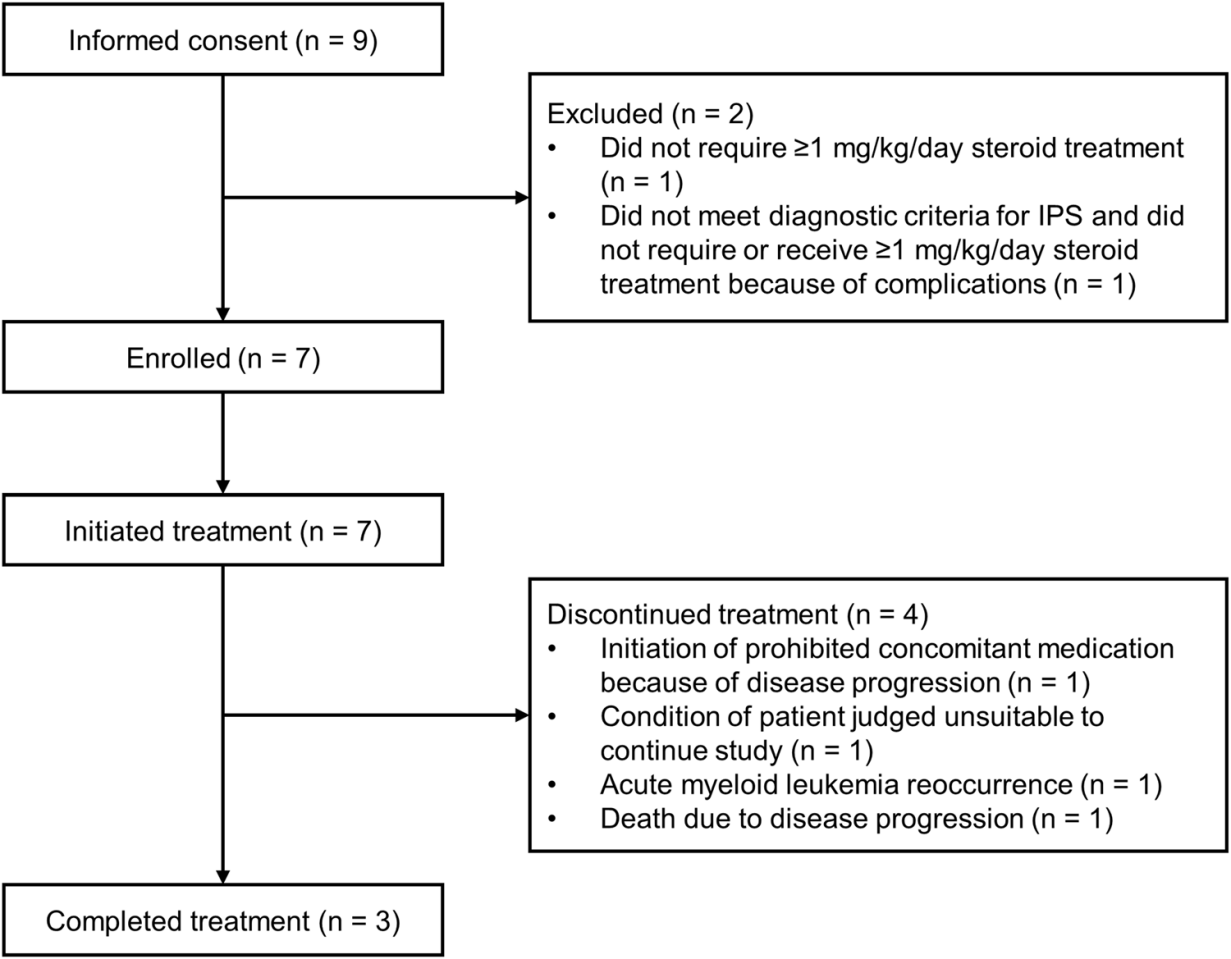
Patient disposition. IPS, idiopathic pneumonia syndrome

The patient disposition is shown in Fig. 1. Nine patients provided informed consent, and two patients were withdrawn during the screening period as they did not meet the eligibility criteria. Seven patients were enrolled, received HLC-001, and were included in the analysis population. Of these, four withdrew after treatment and three completed the trial.

The full analysis set, per-protocol set, and safety analysis set were identical populations, and the baseline characteristics are shown in Table 2. All patients were male, and the mean ± standard deviation (SD) age of patients was 43.3 ± 14.5 years. The rates of acute myeloid leukemia, acute lymphoblastic leukemia, and non-Hodgkin lymphoma were 57.1% (4/7), 28.6% (2/7), and 14.3% (1/7), respectively.

**Table 2:** Patient characteristics

Characteristic	Patients
Male	7 (100.0)
Age, years	43.3 ± 14.5
	43.0 (21–64)
Height, cm	166.63 ± 5.02
	166.20 (158.0–171.8)
Weight, kg	53.40 ± 6.82
	51.50 (46.1–66.9)
White blood cells, × 10^3^/µL	9.830 ± 5.726
	10.510 (4.20–19.90)
CRP, mg/dL	0.896 ± 0.879
	0.620 (0.05–2.22)
KL-6, U/mL	472.1 ± 289.8
	307.0 (218–971)
SP-D, ng/mL	224.10 ± 247.91
	208.00 (27.8–754.0)
PaO_2_/FiO_2_, mmHg	374.81 ± 133.21
	404.80 (154.0–566.7)
Corticosteroid dose, mg/kg/day	1.44 ± 0.83
	1.28 (0.4–3.0)
Hematopoietic malignancy type	
Acute myeloid leukemia	4 (57.1)
Acute lymphoblastic leukemia	2 (28.6)
Non-Hodgkin lymphoma	1 (14.3)
Pre-treatment conditioning regimen	
Myeloablative conditioning	6 (85.7)
Non-myeloablative conditioning	1 (14.3)
Chemotherapy	
Busulfan	3 (42.9)
Fludarabine	7 (100.0)
Melphalan	3 (42.9)
Other	6 (85.7)
Donor and recipient HLA types	
Mismatched related donor	2 (28.6)
Matched unrelated donor	4 (57.1)
Mismatched unrelated donor	1 (14.3)
Hematopoietic stem cell source	
Peripheral blood	5 (71.4)
Bone marrow	2 (28.6)
Acute GVHD	6 (85.7)
Chronic GVHD	3 (42.9)
Prophylaxis for GVHD	2 (28.6)
ECOG performance status	
1	1 (14.3)
2	4 (57.1)
3	1 (14.3)
4	1 (14.3)
Oxygen therapy	4 (57.1)
Oxygen therapy without mechanical ventilation or non-invasive positive pressure ventilation	3 (42.9)
Mechanical ventilation or non-invasive positive pressure ventilation	1 (14.3)

n = 7, unless otherwise stated. Data are shown as n (%) or mean ± standard deviation and median (range)

*CRP* c-reactive protein, *ECOG* Eastern Cooperative Oncology Group, *FiO*_*2*_ fraction of inspired oxygen, *GVHD* graft-versus-host disease, *HLA* human leukocyte antigen, *KL-6* Krebs von den Lungen-6, *PaO*_*2*_ partial pressure of oxygen in arterial blood, *SP-D* surfactant protein-D, *SpO*_*2*_ saturation of peripheral oxygen

The types of conditioning regimen were myeloablative conditioning in 85.7% (6/7) of patients and non-myeloablative conditioning in 14.3% (1/7). The donor and recipient human leukocyte antigen types were mismatched related donor in 28.6% (2/7) of patients, matched unrelated donor in 57.1% (4/7), and mismatched unrelated donor in 14.3% (1/7). The hematopoietic stem cell source was peripheral blood in 71.4% (5/7) of patients and bone marrow in 28.6% (2/7). Six (85.7%) patients developed acute GVHD between HSCT and their participation in the trial. Three (42.9%) patients developed chronic GVHD between HSCT and their participation in the trial. Three (42.9%) patients were administered oxygen without mechanical ventilation or non-invasive positive pressure ventilation, and one (14.3%) patient underwent mechanical ventilation.

The median number of HLC-001 doses was 6 (range: 4–8), and the median total dose was 640.0 × 10^6^ cells (range: 368–1064 × 10^6^ cells). All patients (7/7) completed two cycles of treatment. For patients who received additional doses, 57.1% (4/7) of patients received treatment up to the fifth administration (cycle 3), 57.1% (4/7) received up to the sixth administration (cycle 3), 42.9% (3/7) received up to the seventh administration (cycle 4), and 28.6% (2/7) received up to the eighth administration (cycle 4). After IPS was evaluated as steroid-dependent or refractory, HLC-001 was administered on the same day or the following day for all subjects.

### Overall survival

The survival rate at day 56 (primary endpoint) was 71.4% (5/7) (95% CI: 29.0%–96.3%). As the primary endpoint was met in at least five patients, the trial was terminated at the end of the final observation of the seven patients, which suggested that HLC-001 was more effective than the existing therapy, according to the TS design.

On day 100, the survival rate remained 71.4% (5/7) (95% CI: 29.0%–96.3%). Among the five patients evaluated for survival after 100 days of treatment, three deaths occurred (caused by worsening IPS, relapse of acute myeloid leukemia, and an unknown cause). The total number of days of survival for these patients was 155, 204, and 246 days, respectively (Table 3).

**Table 3:** Individual patient backgrounds and outcomes

	Patient 1	Patient 2	Patient 3	Patient 4	Patient 5	Patient 6	Patient 7
Age	64	21	43	49	56	36	34
Hematopoietic malignancy type	AML	Acute lymphoblastic leukemia	AML	AML	Acute lymphoblastic leukemia	AML	Non-Hodgkin lymphoma
Pre-treatment conditioning regimen (TBI)	Myeloablative procedures (No)	Myeloablative procedures (12 Gy)	Myeloablative procedures (4 Gy)	Myeloablative procedures (4 Gy)	Myeloablative procedures (4 Gy)	Myeloablative procedures (4 Gy)	Non-myeloablative procedures (2 Gy)
Hematopoietic stem cell source	Peripheral blood	Peripheral blood	Bone marrow	Peripheral blood	Bone marrow	Peripheral blood	Peripheral blood
Donor and recipient HLA types	Matched unrelated donor	Mismatched related donor	Matched unrelated donor	Mismatched related donor	Mismatched unrelated donor	Matched unrelated donor	Matched unrelated donor
Time from HSCT to HLC-001 administration, days	109	158	148	239	189	128	687
Acute GVHD	Yes^a^	Yes	Yes	Yes	Yes	No	Yes
Chronic GVHD	No	No	Yes	Yes^a^	No	No	Yes^a^
Corticosteroid dose^b^ (mg/kg/day)	25.1	27.3	1.3	≧ 1.0	1.1	1.3	1.0
P/F ratio	154	413	566.7	382.1	258.3	404.8	444.8
Oxygen therapy	Yes	Yes	No	Yes	Yes	No	No
Initial oxygen dose	With mechanical ventilation	30 L/min	–	2 L/min	4 L/min	–	–
Mechanical ventilation	Yes	No	No	No	No	No	No
Outcome	Died on day 50 (IPS)	Died on day 35 (IPS)	Died on day 204 (relapsed AML)	Died on day 246 (unknown cause)	Died on day 155 (IPS)	Alive on day 485	Alive on day 729

*AML* acute myeloid leukemia, *GVHD* graft-versus-host disease, *HLA* human leukocyte antigen, *HSCT* hematopoietic stem cell transplantation, *IPS* idiopathic pneumonia syndrome, *P/F ratio* partial pressure of oxygen in arterial blood (PaO_2_) and fraction of inspired oxygen (FiO_2_) ratio, *TBI* total body irradiation

^a^Acute or chronic GVHD was observed at the time of HLC-001 administration

^b^Maximum dose (prednisolone or equivalent) before HLC-001 administration

### Mechanical ventilation and oxygen treatment

The percentage of patients who were not receiving mechanical ventilation was 85.7% (6/7) at all evaluated time periods (95% CI: 42.1%–99.6%). The mean ± SD number of days during which mechanical ventilation was not used at days 28, 56, and 100 were 24.0 ± 10.6 days, 44.9 ± 21.2 days, and 65.9 ± 38.6 days, respectively (Table 4).

**Table 4:** Days without mechanical ventilation

Assessment period	Days without mechanical ventilation
Day 0 to day 28	24.0 ± 10.6
	28.0 (0–28)
Day 0 to day 56	44.9 ± 21.2
	56.0 (0–56)
Day 0 to day 100	65.9 ± 38.6
	71.0 (0–100)

n = 7. Data are shown as mean ± standard deviation, or median (range). Data from patients who died or discontinued before each evaluation time period were imputed using the data up to the time of death or discontinuation

The percentages of patients who did not require an increase in oxygen dose on days 28, 56, and 100 were 85.7% (6/7) (95% CI: 42.1%–99.6%), 71.4% (5/7) (95% CI: 29.0%–96.3%), and 71.4% (5/7) (95% CI: 29.0%–96.3%), respectively.

The percentage of patients who did not require oxygen administration was 57.1% (4/7) (95% CI: 18.4%–90.1%) on days 28, 56, and 100. Among patients who required oxygen administration (3/7), the percentages of patients who had oxygen treatment reduced by ≥ 50% on days 28, 56, and 100 were 100.0% (3/3) (95% CI: 29.2%–100.0%), 66.7% (2/3) (95% CI: 9.4%–99.2%), and 66.7% (2/3) (95% CI: 9.4%–99.2%), respectively.

The mean ± SD number of days on which an increase in oxygen dose was not required (n = 7) were 24.0 ± 10.6 days, 44.3 ± 21.6 days, and 65.3 ± 39.1 days at days 28, 56, and 100, respectively. The mean ± SD number of days on which oxygen administration was not required were 15.4 ± 14.5 days, 31.4 ± 29.4 days, and 52.4 ± 50.0 days at days 28, 56, and 100, respectively. Among patients who required oxygen administration (n = 3), the mean ± SD number of days for which a ≥ 50% reduction in oxygen dose was possible were 19.3 ± 9.9 days, 34.0 ± 15.7 days, and 48.7 ± 41.0 days on days 28, 56, and 100, respectively.

### Corticosteroid dose

The mean ± SD corticosteroid dose was 1.2 ± 1.9 mg/kg/day, and the mean ± SD total corticosteroid dose was 2607.5 ± 3310.2 mg. The percentage of patients who were able to reduce their corticosteroid dose to < 1 mg/kg/day was 83.3% (5/6) (95% CI: 35.9%–99.6%). One patient was not included in this analysis because the daily corticosteroid dose was < 1 mg/kg/day at treatment initiation. The mean ± SD reduction in corticosteroid dose was 0.96 ± 0.42 mg/kg.

### Oxygen saturation

**Fig. 2:**
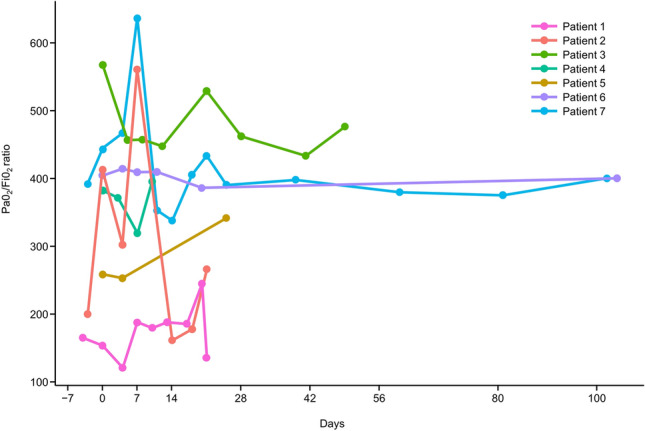
Partial pressure of oxygen in arterial blood and fraction of inspired oxygen ratio over time. Change over time in the ratio of partial pressure of oxygen in arterial blood and fraction of inspired oxygen of HLC-001-treated patients with idiopathic pneumonia syndrome after hematopoietic stem cell transplantation. PaO_2_, partial pressure of oxygen in arterial blood; FiO_2_, fraction of inspired oxygen

The PaO_2_/FiO_2_ ratio is shown in Fig. 2. A temporary improvement in PaO_2_/FiO_2_ ratio was observed for two patients (Patient 2 and Patient 7) during the early stages of administration. Although overall fluctuations in values were noted, corresponding to the patients’ conditions, no cases showed a marked worsening in PaO_2_/FiO_2_ during the evaluation period.

### Imaging results and ECOG-PS scores

Improvements were seen on chest X-ray images in 66.7% (4/6) of patients on day 28 and 60.0% (3/5) of patients on day 56. At day 100, 33.3% (1/3) of patients had improvements on their chest x-ray images and 66.7% (2/3) had no changes.

Regarding chest CT images, improvements were seen in 66.7% (4/6) of patients on day 28 and 40.0% (2/5) of patients on day 56. At day 100, 66.7% (2/3) of patients had improvements on their CT images and 33.3% (1/3) had no changes.

**Fig. 3:**
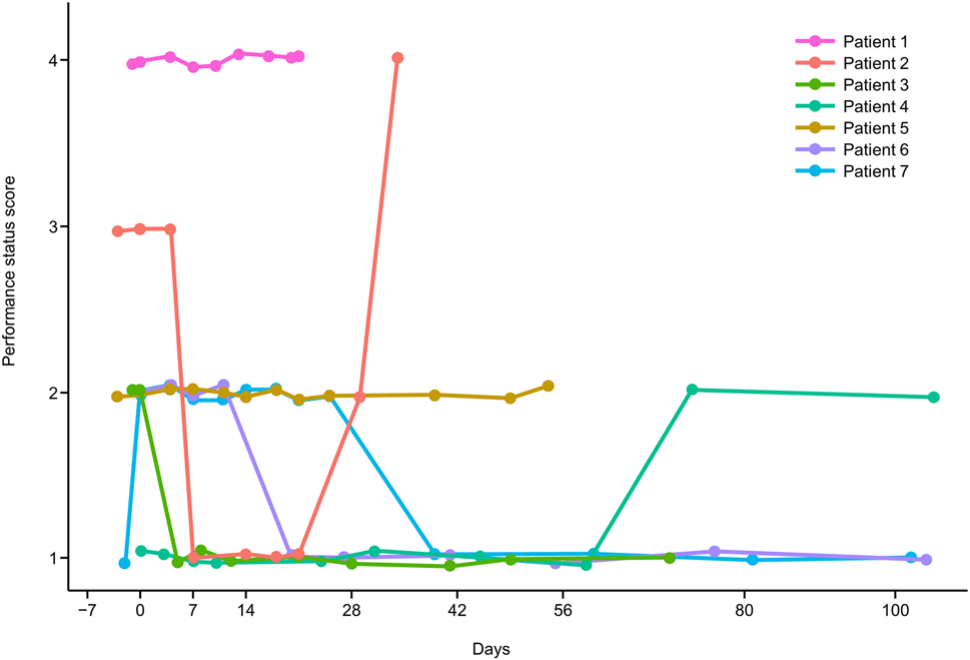
Eastern Cooperative Oncology Group performance status scores^a^ over time. Change over time in Eastern Cooperative Oncology Group performance status scores of HLC-001-treated patients with idiopathic pneumonia syndrome after hematopoietic stem cell transplantation. ^a^0 = Fully active, able to carry on all pre-disease performance without restriction; 1 = Restricted in physically strenuous activity but ambulatory and able to carry out work of a light or sedentary nature, e.g., light housework, office work; 2 = Ambulatory and capable of all selfcare but unable to carry out any work activities, up and about more than 50% of waking hours; 3 = Capable of only limited selfcare, confined to bed or chair more than 50% of waking hours; 4 = Completely disabled, cannot carry out any selfcare, totally confined to bed or chair

Three patients had improved ECOG-PS scores over time (Patients 3, 6, and 7), and two patients had no change in ECOG-PS scores (Patients 1 and 5). Worsened ECOG-PS scores were observed in two patients (Patients 2 and 4). For one patient (Patient 2), the ECOG-PS score temporarily improved and then deteriorated at treatment discontinuation (Fig. 3).

### Serum biomarkers

**Fig. 4:**
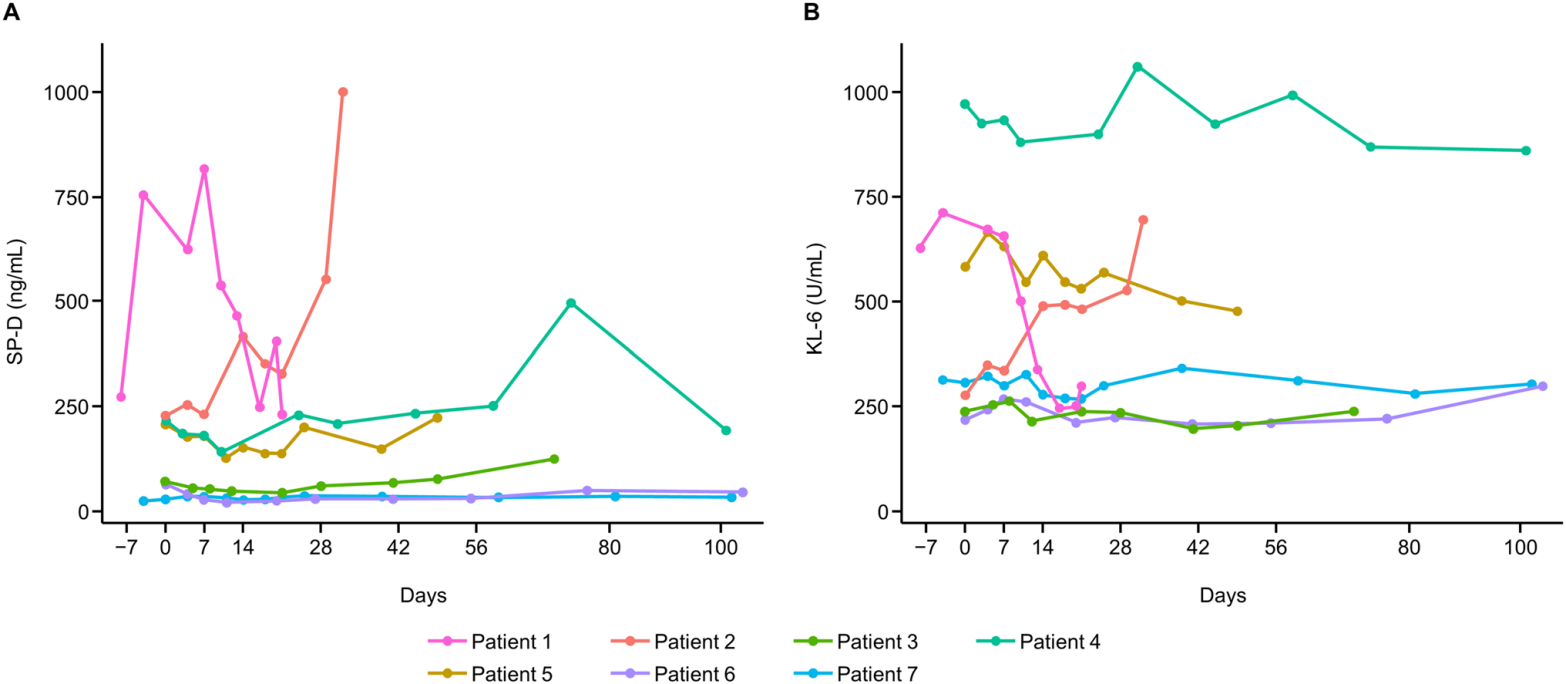
SP-D and KL-6 levels over time. Change over time in (**A**) SP-D levels and (**B**) KL-6 levels of HLC-001-treated patients with idiopathic pneumonia syndrome after hematopoietic stem cell transplantation. SP-D, surfactant protein-D; KL-6, Krebs von den Lungen-6

Changes in SP-D and KL-6 levels over time are shown in Fig. 4. SP-D and KL-6 levels increased over time in one patient (Patient 2) and decreased over time in another patient (Patient 1). There were no notable changes over time observed in the other patients.

### Safety

All patients experienced AEs, and a total of 57 AEs were reported (Table 5). A total of 30 adverse drug reactions (ADRs) were reported, and 71.4% (5/7) of patients experienced at least one ADR (Table S2). No AEs of Grade 4 or 5 severity were observed. Regarding Grade 3 events, there were eight infusion-related events (14.3%) (1/7 patients), four pneumothorax events (28.6%) (2/7 patients), one hyperglycemia (14.3%), one pneumonia (14.3%), one COVID-19 infection (14.3%), one decreased platelet count event (14.3%), and one hypertension event (14.3%). Of these, the infusion-related reactions and hypertension were judged to be ADRs. Six serious AEs occurred in 57.1% (4/7) of patients (four pneumothorax events in two [28.6%] patients, one COVID-19 infection in one [14.3%] patient, and one recurrent acute myeloid leukemia event in one [14.3%] patient). All serious AEs were considered unrelated to HLC-001 treatment.

**Table 5:** Adverse events

Event	Number of events	Patients
Any adverse events	57	7 (100.0)
Metabolism and nutrition disorders	4	4 (57.1)
Hypokalemia	2	2 (28.6)
Hyperglycemia	1	1 (14.3)
Hyperuricemia	1	1 (14.3)
Respiratory, thoracic and mediastinal disorders	5	3 (42.9)
Pneumothorax	4	2 (28.6)
Pneumomediastinum	1	1 (14.3)
Vascular disorders	6	3 (42.9)
Hypotension	4	1 (14.3)
Hypertension	1	1 (14.3)
Phlebitis	1	1 (14.3)
General disorders and administration site conditions	4	3 (42.9)
Pyrexia	2	2 (28.6)
Catheter site pain	1	1 (14.3)
Edema	1	1 (14.3)
Infections and infestations	3	3 (42.9)
Pneumonia	2	2 (28.6)
COVID-19	1	1 (14.3)
Injury, poisoning and procedural complications	11	2 (28.6)
Infusion-related reaction	8	1 (14.3)
Procedural pain	3	1 (14.3)
Nervous system disorders	8	2 (28.6)
Headache	4	1 (14.3)
Head discomfort	4	1 (14.3)
Cardiac disorders	2	2 (28.6)
Palpitations	1	1 (14.3)
Sinus tachycardia	1	1 (14.3)
Psychiatric disorders	2	2 (28.6)
Insomnia	2	2 (28.6)
Skin and subcutaneous tissue disorders	2	2 (28.6)
Rash	1	1 (14.3)
Skin exfoliation	1	1 (14.3)
Investigations	5	1 (14.3)
Oxygen saturation decreased	4	1 (14.3)
Platelet count decreased	1	1 (14.3)
Blood and lymphatic system disorders	1	1 (14.3)
Thrombotic microangiopathy	1	1 (14.3)
Ear and labyrinth disorders	1	1 (14.3)
Ear discomfort	1	1 (14.3)
Gastrointestinal disorders	1	1 (14.3)
Hematochezia	1	1 (14.3)
Immune system disorders	1	1 (14.3)
Seasonal allergy	1	1 (14.3)
Neoplasms benign, malignant and unspecified (including cysts and polyps)	1	1 (14.3)
Recurrent acute myeloid leukemia	1	1 (14.3)

n = 7. Data are shown as n or n (%). Events are described by System Organ Class and Preferred Term according to the Medical Dictionary for Regulatory Activities, Japanese version 26.0

### Exploratory endpoints: cytokines, chemokines, and cell assessments

As an exploratory study, an examination of blood cytokines, chemokines, and immune cells was conducted in five patients. These parameters varied among the patients based on their conditions and the number of doses of HLC-001 administered, making it difficult to identify consistent trends. However, natural killer (NK) cell counts tended to increase up to day 28, except for one patient (Patient 2), who died on day 35 due to worsened IPS.

## Discussion

IPS is a rare and fatal non-infectious pulmonary complication occurring after HSCT. It is characterized by extremely high short-term mortality and a poor prognosis. Steroid therapy is commonly administered, but the response rate is poor, and there is no established treatment, highlighting the need for developing new therapies. In this clinical trial, the efficacy and safety of HLC-001, an allogeneic umbilical cord-derived MSC, were evaluated as a potential treatment for IPS.

The primary endpoint of this clinical trial was the overall survival rate at day 56 following the initiation of HLC-001 administration. In a frequentist fixed sample size design, the anticipated survival probability at day 56 after the onset of IPS due to HLC-001 would need to be pre-specified, and the corresponding sample size required to detect a significant difference would be estimated. However, there were insufficient data to predict the magnitude of the effect that HLC-001 might have in comparison to existing treatments. Furthermore, when calculating the required sample size based on frequentist methods, a substantially larger sample would have been necessary, which, given the rarity of IPS, was deemed impractical. A key advantage of utilizing a TS design is that it obviates the need to predefine a precise expected survival probability for the study treatment at day 56 after the onset of IPS. Additionally, it is expected that the required sample size to achieve comparable power would be smaller than that of a frequentist fixed sample size design. Moreover, the results of the trial can be summarized as a posterior distribution, allowing for robust inferences within the framework of the prior distribution, even in cases where statistical significance is not achieved, in contrast to the hypothesis testing framework of frequentist designs. For these reasons, we selected a TS design for this phase II trial.

In this study, five of the seven patients who received HLC-001 were alive 56 days after the start of treatment (overall survival rate: 71.4% [95% CI: 29.0%–96.3%]). Based on the TS design, this suggests that HLC-001 was more effective than the existing treatments. In the TS design, the survival probability at 56 days after the onset of IPS with existing treatments was estimated to be 24.5% based on a mathematical integration of results from 126 cases [3–5]. The 95% CI for the overall survival rate at day 56 after HLC-001 administration exceeded this 24.5% estimate for existing treatments. Furthermore, the survival rate at day 100 after administration remained comparable to that at day 56, indicating sustained survival up to day 100 after administration. According to other studies, approximately 80% of patients with IPS die within 100 days of onset [2–5]. When compared to these studies, HLC-001 appeared to improve survival rates up to 100 days after IPS onset. Of the patients in our study who we were able to observe past 100 days, there were three deaths within a year of HLC-001 administration, attributed to worsening of IPS (Patient 5), relapse of acute myeloid leukemia (Patient 3), and an unknown cause (Patient 4). The 1-year survival rate after HLC-001 administration was 28.6% (2 out of 7 cases). Literature reports indicate that deaths due to causes other than worsening of IPS increase after 100 days from onset [5, 17]. Given the rapid progression and high mortality rate for IPS within the first 100 days of symptom onset, improving survival rates up to this time point is clinically significant, as deaths due to causes other than worsening of IPS increase after 100 days from onset.

In this study, the need for mechanical ventilation among patients was low, with only one patient requiring mechanical ventilation during the trial. Additionally, four of the seven patients (57.1%) did not require oxygen administration on days 28, 56, or 100. Patients with IPS who require mechanical ventilation have a poor prognosis, with a reported 90% mortality rate [7, 18]. Given that most patients in this trial did not require mechanical ventilation or an increase in oxygen support, HLC-001 may help prevent the progression of IPS and reduce the need for mechanical ventilation. Moreover, the steroid dose was reduced to < 1 mg/kg in five out of six patients, and most patients who were still alive at day 100 showed either improvements or stability in PaO_2_/FiO_2_, imaging findings, PS scores, SP-D, and KL-6 levels. Together, these findings suggest that HLC-001 may help prevent progression of IPS.

In the safety evaluation of HLC-001, all patients experienced AEs and 71.4% of patients experienced ADRs. There were no Grade 4 or 5 events. Grade 3 events included infusion-related reactions (eight events; one patient), pneumothorax (four events; two patients), and one event each of hyperglycemia, pneumonia, COVID-19, decreased platelet count, and hypertension. Both the infusion-related reactions and hypertension were considered related to the study drug. The infusion-related reactions occurred in one patient on the day of HLC-001 administration, and this was managed by adjusting the infusion rate and temporarily suspending drug administration. Hypertension occurred in one patient 14 days after the first administration of HLC-001; however, it was not possible to follow up on the outcome as the patient subsequently died due to worsening of IPS. The serious AEs were pneumothorax in two patients, COVID-19 (one patient), and relapsed acute myeloid leukemia (one patient), but no serious AEs were related to HLC-001. As an important point, all AEs were manageable, suggesting HLC-001 is generally well tolerated in patients with IPS.

Additionally, exploratory studies were conducted to measure blood cytokines, chemokines, and immune cells. In the patients who were alive at day 100, there was a trend towards an increase in NK cells. This is consistent with the results of a phase I trial of HLC-001 for acute GVHD [13], which also reported a trend towards increased NK cell count in the patients who responded to treatment. Although the clinical significance of this increase in NK cell count in patients who responded favorably to HLC-001 is unknown, it is a very interesting finding and further research is expected in the future.

This trial had some limitations, including the small population size (seven patients). Additionally, all patients included in this trial were male; therefore, any potential sex differences could not be evaluated. In this clinical trial, only patients capable of receiving an explanation and providing informed consent were enrolled. Finally, the use of BAL at study entry could have influenced disease severity. When compared with retrospective studies and data, these factors could have potentially introduced selection bias. Despite these limitations, the reported findings are clinically important as steroid-dependent and refractory IPS currently has no cure.

In conclusion, this trial found that HLC-001 treatment was effective and generally well tolerated in Japanese patients with steroid-dependent or refractory IPS after HSCT. As there is no effective cure for IPS once it becomes steroid-dependent or resistant, our findings indicate that HLC-001 may be a promising treatment option for affected patients; however, further clinical evaluation is warranted to gather more robust evidence supporting its efficacy and safety.

## Supplementary Information

Below is the link to the electronic supplementary material.

Supplementary file1 (DOCX 40 KB)

## Data Availability

The data that support the findings of the study can be made available from the corresponding author or the study sponsor upon reasonable request.
