# Supplementary material for: Intravenous umbilical cord-derived mesenchymal stromal cell therapy may improve overall survival in Japanese patients with idiopathic pneumonia syndrome after hematopoietic stem cell transplantation: a multicenter, single-arm, phase II trial
Source: Int J Hematol. 2025 Jul 15;122(5):733–43. doi: 10.1007/s12185-025-04024-x (PMC12572052; doi:10.1007/s12185-025-04024-x)
Supplement: Supplementary file 1 — Supplementary file1 (DOCX 40 KB) [file 12185_2025_4024_MOESM1_ESM.docx]

**Supplementary materials**

**Supplementary methods**

*Study design and treatment*

The study period was from the day of informed consent to the last observation day (day 100 after treatment administration or the discontinuation day), and included the screening period, treatment period, and observation period. The screening period was 14 days prior to administration. The observation period followed the completion of the treatment period and lasted until the last observation day.

During the treatment period, patients received HLC-001 intravenously at a dose of 2 × 10^6^ cells/kg of body weight, once daily, for two doses per cycle at intervals between 2 and 7 days. Patients received two cycles (four doses total), and, if safety was confirmed and the response was inadequate, patients could receive up to two additional cycles (i.e., in total, a maximum of four cycles and eight doses).

An independent Effectiveness and Safety Evaluation Committee of four experts in hematopoietic stem cell transplantation, respiratory medicine, intensive care, and radiation was established. This committee discussed the safety of all patients and the diagnosis of idiopathic pneumonia syndrome (IPS).

*Exclusion criteria*

The exclusion criteria were: active pulmonary infection; heart failure that may contribute to respiratory failure; current or prior treatment with bone marrow-derived mesenchymal stromal cell preparations (Temcell^®^ HS injection) or Bruton’s tyrosine kinase inhibitor (Imburvica^®^ capsule [Ibrutinib; Janssen Biotech, Inc., USA, and Pharmacyclics LLC, USA]); requiring new systemic immunosuppressants or corticosteroid dose increases (except as-needed for allergic reactions); treatment for malignancy other than hematopoietic stem cell transplantation within 3 months of enrollment, or completed treatment by 3 months of enrollment but with an unstable malignancy; previous acute myocardial infarction within 6 months of enrollment; history of primary pulmonary hypertension and complications; renal dysfunction requiring dialysis; total serum bilirubin ≥3 × upper limit of normal (ULN), aspartate aminotransferase and alanine aminotransferase ≥5 × ULN, or serum creatinine level ≥2 × ULN of the reference level of the study site within 14 days of enrollment (excluding values caused by GVHD); allergy to amphotericin B or gentamicin; previous emergency treatment because of an adverse reaction to dimethyl sulfoxide; positive for HIV antibody, human T-cell leukemia virus-1 antibody, hepatitis B surface antigen, hepatitis B core antibody, hepatitis B surface antibody, or hepatitis C antibody within 30 days before registration (except for hepatitis B surface antibody caused by vaccination or HIV infection considered adequately controlled by the investigator); pregnant, lactating, or possibly pregnant; participation in other trials; administration of another investigational or unapproved medication; or judged by the investigator or sub-investigator to be inappropriate for participation in the trial.

*Assessments*

Survival was assessed on days 21, 28, 42, 56, 80, and 100. Survival verification surveys were conducted 6 months and 1 year after the end of the last observation in the last case. Imaging results were evaluated as “improved”, “unchanged”, or “aggravated” when compared with the findings in the screening period. The background assessments included patient age, height, weight, systolic and diastolic blood pressure, pulse, body temperature, saturation of percutaneous oxygen, respiratory rate, white blood cell count, c-reactive protein, Krebs von den Lungen-6, surfactant protein-D, partial pressure of oxygen in arterial blood (PaO_2_), partial pressure of carbon dioxide in arterial blood, PaO_2_ and fraction of inspired oxygen ratio, steroid dose, type of hematopoietic malignancy, conditioning regimen, donor and recipient HLA types, source of hematopoietic stem cell transplantation, presence of acute or chronic GVHD, GVHD prophylaxis, Eastern Cooperative Oncology Group performance status scores, and use of oxygen therapy, mechanical ventilation, or non-invasive positive pressure ventilation.

The biomarkers evaluated as exploratory assessments included CD3^−^CD16^+^CD56^+^ cells; CD3^−^CD19^+^ cells; CD3^+^ cells; CD3^+^CD4^+^ cells; CD3^+^CD8^+^ cells; cutaneous T-cell attracting chemokine; CXCL6; eotaxin; basic fibroblast growth factor-2; granulocyte colony stimulating factor; granulocyte-macrophage colony stimulating factor; GRO-α; hepatocyte growth factor, interferon (IFN)-α2; IFN-γ; interleukin (IL)-10; IL-12(p40); IL-12(p70); IL-13; IL-15; IL-16; IL-17; IL-18; IL-1RA; IL-1α; IL-1β; IL-2; IL-2Rα; IL-3; IL-4; IL-5; IL-6; IL-7; IL-8; IL-9; IFN-γ inducible protein of 10 kDa, leukemia inhibitory factor; macrophage colony stimulating factor; monocyte chemoattractant protein-1; monocyte chemotactic protein-3; macrophage migration inhibitory factor; monokine induced by IFN-γ; macrophage inflammatory protein-1α; macrophage inflammatory protein-1β; platelet-derived growth factor-BB; regulated upon activation, normal T-cell expressed and secreted (RANTES); stem cell factor; SOGF-β, stromal cell-derived factor-1α, tumor necrosis factor (TNF)-α, TNF-β, TNF-related apoptosis-inducing ligand; vascular endothelial growth factor; and β-nerve growth factor.

*Statistical methods*

The Bayesian design by Thall & Simon (1994) (TS design)^1^ was used for this trial. For a given total number of treatments (n = 1, 2, 3, up to n = max), the probability of survival at 56 days post-IPS onset for the trial product (P_E_) and the existing treatment (P_S_) was assumed. Patient enrollment was adaptively determined based on the posterior probability Pr (P_E_ > P_S_│y, n) according to the following rules: if the enrolled number of patients was ≥6 and Pr (P_E_ > P_S_│y, n) ≥0.95, the trial product was considered more effective. If n ≥ 6 and Pr (P_E_ > P_S_│y, n) ≤0.05, the trial product was not considered more effective. If neither condition was met, enrollment continued. Patients with missing survival data at 56 days post-IPS diagnosis were excluded from the total treatment count, with decisions following the TS design rules. The prior probability P_E_–Beta (α_E_ = 0.2, β_E_ = 1.8) was selected as uninformative prior based on the results of the operational characteristics, which are consistent with frequentist hypothesis testing. The survival probability at 56 days after the onset of IPS under existing treatments was estimated to be 0.2453 by mathematically integrating the results of 126 cases reported in previous studies^2–4^. Based on this estimation, a beta distribution with parameters α = 30.91 and β = 95.09 was used.

Previous studies were selected by searching PubMed with a focus on literature published between January 1, 2000, and December 31, 2021. The selected studies included those that diagnosed IPS using the definition by the National Heart, Lung, and Blood Institute consensus or the American Thoracic Society statement, and contained data on survival after the onset of IPS. Additionally, studies that described the implementation of bronchoalveolar lavage were selected.

*References*

1. Thall PF, Simon R. Practical Bayesian guidelines for phase IIB clinical trials. Biometrics. 1994; 50(2):337-49.

2. Fukuda T, Hackman RC, Guthrie KA, Sandmaier BM, Boeckh M, Maris MB, et al. Risks and outcomes of idiopathic pneumonia syndrome after nonmyeloablative and conventional conditioning regimens for allogeneic hematopoietic stem cell transplantation. Blood. 2003; 102(8):2777-85.

3. Zhu KE, Hu JY, Zhang T, Chen J, Zhong J, Lu YH. Incidence, risks, and outcome of idiopathic pneumonia syndrome early after allogeneic hematopoietic stem cell transplantation. Eur J Haematol. 2008; 81(6):461-6.

4. Tizon R, Frey N, Heitjan DF, Tan KS, Goldstein SC, Hexner EO, et al. High-dose corticosteroids with or without etanercept for the treatment of idiopathic pneumonia syndrome after allo-SCT. Bone Marrow Transplant. 2012; 47(10):1332-7.

**Table S1.** Participating institutions and representative physicians

| **Institution** | **Physician** |
| --- | --- |
| Tokyo Metropolitan Komagome Hospital | Noriko Doki |
| Okayama University Hospital | Nobuharu Fujii |
| Jichi Medical University Saitama Medical Center | Shinichi Kako |
| Chiba University Hospital | Emiko Sakaida |
| Jichi Medical University Hospital | Yoshinobu Kanda |

**Table S2.** Adverse drug reactions

| **System Organ Class and Preferred Term^a^** | **Number of events** | **Patients** |
| --- | --- | --- |
| Adverse drug reactions | 30 | 5 (71.4) |
| Nervous system disorders | 8 | 2 (28.6) |
| Headache | 4 | 1 (14.3) |
| Head discomfort | 4 | 1 (14.3) |
| Injury, poisoning and procedural complications | 8 | 1 (14.3) |
| Infusion related reaction | 8 | 1 (14.3) |
| Vascular disorders | 6 | 3 (42.9) |
| Hypotension | 4 | 1 (14.3) |
| Hypertension | 1 | 1 (14.3) |
| Phlebitis | 1 | 1 (14.3) |
| Investigations | 4 | 1 (14.3) |
| Oxygen saturation decreased | 4 | 1 (14.3) |
| Blood and lymphatic system disorders | 1 | 1 (14.3) |
| Thrombotic microangiopathy | 1 | 1 (14.3) |
| Cardiac disorders | 1 | 1 (14.3) |
| Palpitations | 1 | 1 (14.3) |
| General disorders and administration site conditions | 1 | 1 (14.3) |
| Pyrexia | 1 | 1 (14.3) |
| Infections and infestations | 1 | 1 (14.3) |
| Pneumonia | 1 | 1 (14.3) |

n = 7. Data are shown as n or n (%).

^a^Medical Dictionary for Regulatory Activities, Japanese version 26.0
